# Supplementary material for: De novo assembled mitogenome analysis of Trichuris trichiura from Korean individuals using nanopore-based long-read sequencing technology
Source: PLoS Negl Trop Dis. 2023 Aug 28;17(8):e0011586. doi: 10.1371/journal.pntd.0011586 (PMC10491297; doi:10.1371/journal.pntd.0011586)
Supplement: S1 Table — (DOCX) [file pntd.0011586.s001.docx]

**S1 Table. Raw reads statistics.** *Raw reads* are the content of FASTQ file output of the ONT sequencing while *Extracted reads* are those that specifically span the mitochondrial contig of the reference complete genome of *T. trichiura* after mapping then were utilized for mitogenome assembly.

| **Statistics** | **TTK1** | **TTK2** | **TTK3** |
| --- | --- | --- | --- |
| Number of raw sequencing reads | 9173 | 15,128 | 5651 |
| Total base pairs (bp) raw data | 19,824,561 | 13,175,884 | 15,867,001 |
| N50 contig size | 4234 | 1246 | 3430 |
| Number of extracted reads | 399 | 1006 | 249 |
| Total base pairs (bp) extracted data | 1,103,615 | 815,543 | 741,548 |
| N50 contig size | 4412 | 1034 | 249 |
| Assembly Coverage | 74X | 30X | 52X |
