# Supplementary material for: De novo assembled mitogenome analysis of Trichuris trichiura from Korean individuals using nanopore-based long-read sequencing technology
Source: PLoS Negl Trop Dis. 2023 Aug 28;17(8):e0011586. doi: 10.1371/journal.pntd.0011586 (PMC10491297; doi:10.1371/journal.pntd.0011586)
Supplement: S2 Table — (DOCX) [file pntd.0011586.s002.docx]

**S2 Table. Features of annotated protein coding genes our mitogenome and the reference sequences.**

| Gene | Position   (bp length \| amino acid) | | | | | |
| --- | --- | --- | --- | --- | --- | --- |
|  | TTK1 | TTK2 | TTK3 | TTJP | TTCN | TTUG |
| *cox1* | 1-1542   (1545 \| 515) | 1-1542   (1545 \| 515) | 1-1542   (1545 \| 515) | 4-1528  (1545 \| 515) | 1-1545  (1545 \| 515) | 1-1545  (1545 \| 515) |
| *cox2* | 1557-2234 (678 \| 225) | 1560-2234 (675 \| 224) | 1560-2234 (675 \| 224) | 1560-2337 (678 \| 225) | 1560-2234 (675 \| 224) | 1558-2232 (675 \| 225) |
| *nd1* | 2397-3296 (900 \| 299) | 2397-3296 (900 \| 299) | 2397-3296 (900 \| 299) | 2400-3299 (900 \| 300) | 2397-3296 (900 \| 299) | 2406-3305 (900 \| 299) |
| *nd2* | 3985-4881 (897 \| 299) | 3920-4804 (885 \| 294) | 3916-4800 (885 \| 295) | 3563-4459 (897 \| 289) | 3522-4406 (885 \| 294) | 3488-4384 (897 \| 298) |
| *nd5* | 4994-6538 (1545 \| 512) | 4929-6476 (1548 \| 515) | 4925-6472 (1548 \| 515) | 4572-6116 (1545 \| 514) | 4531-6078 (1548 \| 516) | 4488-6044 (1557 \| 518) |
| *nd4* | 6658-7893 (1236 \| 412) | 6593-7804 (1212 \| 403) | 6589-7800 (1212 \| 404) | 6236-7471 (1236 \| 411) | 6195-7406 (1212 \| 403) | 6165-7382 (1218 \| 405) |
| *nd4L* | 7902-8150 (249 \| 82) | 7823-8080 (258 \| 85) | 7819-8076 (258 \| 86) | 7480-7728 (249 \| 83) | 7425-7682 (258 \| 85) | 7405-7617 (213 \| 71) |
| *nd6* | 8272-8739 (468 \| 156) | 8193-8669 (477 \| 158) | 8327-8755 (429 \| 158) | 7850-8317 (468 \| 155) | 7795-8271 (477 \| 159) | 7766-8242 (477 \| 158) |
| *cytb* | 8740-9852 (1113 \| 371) | 8676-9782 (1107 \| 368) | 8672-9778 (1107 \| 368) | 8318-9430 (1113 \| 370) | 8278-9384 (1107 \| 369) | 8249-9355 (1107 \| 368) |
| *atp6* | 11705-12440 (736 \| 244) | 11570-12397 (828 \| 275) | 11566-12393 (828 \| 276 | 11286-12021 (736 \| 245) | 11173-12000 (828 \| 276) | 11141-11953 (813 \| 270) |
| *cox3* | 12441-13214 (774 \| 257) | 12372-13145 (774 \| 257) | 12368-13141 (774 \| 257) | 12022-12795 (774 \| 258) | 11975-12748 (774 \| 257) | 11959-12723 (774 \| 258) |
| *atp8* | 13533-13685 (153 \| 50) | 13452-13616 (165 \| 54) | 13448-13612 (165 \| 54) | 13115-13267 (153 \| 50) | 13055-13219 (165 \| 54) | 13052-13219 (168 \| 55) |
| *nd3* | 13695-14036 (342 \| 113) | 13626-13967 (342 \| 113) | 13622-13963 (342 \| 113) | 13277-13618 (342 \| 113) | 13229-13570 (342 \| 113) | 13229-13570 (342 \| 113) |
