# Supplementary material for: De novo assembled mitogenome analysis of Trichuris trichiura from Korean individuals using nanopore-based long-read sequencing technology
Source: PLoS Negl Trop Dis. 2023 Aug 28;17(8):e0011586. doi: 10.1371/journal.pntd.0011586 (PMC10491297; doi:10.1371/journal.pntd.0011586)
Supplement: S3 Table — T. trichiura China (GU385218) is used as reference genome for variant calling since it is the closest published reference mitogenome. (DOCX) [file pntd.0011586.s003.docx]

**S3 Table. Snpeff variant calling results.** *T. trichiura* China (GU385218) is used as reference genome for variant calling since it is the closest published reference mitogenome. (Ms – missense, Ns – nonsense, Si – sense mutation)

| Mito  genome | Variants | | | Transitions / Transversions  \| Ratio | Mutations  (count \| %) | | | | Synonymous (dS)/ Nonsynonymous (dN) | | |
| --- | --- | --- | --- | --- | --- | --- | --- | --- | --- | --- | --- |
|  | SNP | INS | DEL |  | Ms | Ns | Si | Ms/Si Ratio | dS | dN | ratio |
| TTK1 | 903 | 6 | 0 | 1,677/116 \| 14.5 | 235 | 17 | 558 | 0.42 | 542 | 1021 | 0.53 |
| TTK2 | 223 | 0 | 1 | 420 /25 \| 16.8 | 74 | 1 | 111 | 0.67 | 109 | 272 | 0.40 |
| TTK3 | 229 | 4 | 4 | 434 /23 \| 18.8 | 0 | 0 | 0 | 0.0 | 0 | 0 | 0 |
