# Supplementary material for: De novo assembled mitogenome analysis of Trichuris trichiura from Korean individuals using nanopore-based long-read sequencing technology
Source: PLoS Negl Trop Dis. 2023 Aug 28;17(8):e0011586. doi: 10.1371/journal.pntd.0011586 (PMC10491297; doi:10.1371/journal.pntd.0011586)
Supplement: S4 Table — Pairwise comparison results between assembled mitogenomes and reference sequences. (DOCX) [file pntd.0011586.s004.docx]

**S4 Table. Variability of mitogenomes.** Pairwise comparison results between assembled mitogenomes and reference sequences. Whole mitogenome, COX1, and ITS1 regions of each mitogenome were extracted and aligned to show variability.

| Reference sequences | *T. trichiura*  Japan | *T. trichiura*  China | *T. trichiura*  Uganda |
| --- | --- | --- | --- |
| Complete mitogenome | | | |
| TtmtK1 | 7.7% | 9.9% | 23.1% |
| TtmtK2 | 18.3% | 14.0% | 30.3% |
| TtmtK3 | 9.0% | 4.4% | 22.4% |
| AP | 0 | 7.1% | 20.7% |
| NC | 7.1% | 0 | 20.2% |
| KT | 20.7% | 20.2% | 0 |
| *cox1* region | | | |
| TtmtK1 | 3.1% | 5.8% | 18.2% |
| TtmtK2 | 5.3% | 5.3% | 16.5% |
| TtmtK3 | 5.1% | 5.1% | 16.5% |
| AP | 0 | 4.8% | 16.8% |
| NC | 4.8% | 0 | 16.4% |
| KT | 16.8% | 16.4% | 0 |
| *ITS* region | | | |
| TtmtK1 | 3.5% | 6.8% | 17.1% |
| TtmtK2 | 7.0% | 1.2% | 17.7% |
| TtmtK3 | 7.5% | 1.7% | 17.7% |
| AP | 0 | 6.3% | 19.9% |
| NC | 6.3% | 0 | 17.4% |
| KT | 19.9% | 17.4% | 0 |
