# Supplementary material for: De novo assembled mitogenome analysis of Trichuris trichiura from Korean individuals using nanopore-based long-read sequencing technology
Source: PLoS Negl Trop Dis. 2023 Aug 28;17(8):e0011586. doi: 10.1371/journal.pntd.0011586 (PMC10491297; doi:10.1371/journal.pntd.0011586)
Supplement: S1 File — (DOCX) [file pntd.0011586.s005.docx]

**S1 File. Scripts**

**Assembly**

#Checking read coverage with whole genome sequence

*samtools coverage -t in.sorted.bam*

*#*Creating SAM file

*minimap2 -ax map-ont whole_genome.fna ONT_raw_zip.fq.gz > output_name.sam*

#Creating BAM file

*samtools view -S -h -b input_name.sam > OUT_name.bam*

#Sorting BAM file

*samtools sort -o output_name.sorted.bam IN_name.bam*

#indexing BAM file

*samtools index IN_name.sorted.bam*

#Checking coverage

*samtools coverage -m IN_name.sorted.bam -r <chr:region>*

#Listing aligned reads

*samtools view -h -o Tt_ont_xtrctd.txt name.sorted.bam chr.1:1-14945*

#Reads extraction

*seqtk subseq ont.fq Tt_ont_xtrctd.txt > output_name_mt.fq*

#*de novo* assembly

*canu genomeSize=14k m -nanopore-raw *ont.reads.fastq.gz*

#Polishing

*minimap2 -ax map-ont in.canu.assembled.fasta in.extracted.reads.fastq > assembled_polished_consensus.sam*

*racon -t 8 in.extracted.reads.fastq assembled_polished_consensus.sam in.canu.assembled.fasta > out.racon.polished.fasta*

#Check output

*assembly-stats -t input_file.fastq*

***Contig scaffolding, polishing, trimming and circularization***

#contig scaffolding

*minimap2 -ax map-ont ref.WG.mt.fasta racon.polished.fasta | samtools sort -O BAM -o racon_mtref_mapped.bam*

#Contig extraction

*samtools view racon_mtref_mapped.bam | grep "contigname" | awk '{print $1}' | sort | uniq > extracted_mt_contig_names.txt*

*seqtk subseq racon.polished.fasta extracted_mt_contig_names.txt > extracted_mt_contig.fasta*

# Trimming 500bp head and tail

*samtools faidx contig_polished.fasta contig_name:region 501-(lastbp-500) > contig_trimmed.fasta*

#circularize mitogenome

*nucmer -maxmatch -nosimplify contig_trimmed.fasta contig_trimmed.fasta*

#Check overhang

*show-coords -lrc out.delta*

#Trim overhang

*samtools faidx contig_trimmed.fasta contig_name:region (except-overhang-position) > circularized_genome.fasta*

*Mitogenome rearrangement and reformatting*

#Arrangement check

n*ucmer -maxmatch -nosimplify circularized_genome.fasta AP017704.1_mtref.fasta*

*show-coords -lrc out.delta*

#Rearrange

*samtools faidx* *circularized_genome.fasta  contig_name:region A-B or B-C  > mitogenome_rearranged.fasta*

# Reformatted to remove the gap in the fasta file and produce a single line of nucleotide sequence

*bioawk -c fastx '{print ">"$name; print $seq}' in.rearranged.fasta > out.rearranged_reformatted.fasta*

**Mitogenome variability heatmap script in R**

#upload csv file of dataset : complete mitogenome variability

View(complete)

x <- read.csv("complete2.csv", row.names = 1)

y <- data.matrix(x)

#set color palette

my_palette <- colorRampPalette(c("yellow", "red"))(n = 50)

#create heatmap

heatmap.2(y, main = "Complete mitogenome", trace = "none",

density.info=c("none"),

margins = c(5,5),

labRow = NULL,

notecex = 1.0,

notecol = "black",

keysize = 1.5,

col =my_palette, dendrogram = c("row"))

# upload csv file of dataset : cox1 mitogenome variability

View(cox1)

x <- read.csv("cox11.csv", row.names = 1)

y <- data.matrix(x)

#set color palette

my_palette <- colorRampPalette(c("yellow", "red"))(n = 50)

#create heatmap

heatmap.2(y, main = "cox1", trace = "none",

density.info=c("none"),

margins = c(5,5),

labRow = NULL,

notecex = 1.0,

notecol = "black",

keysize = 1.5,

col =my_palette, dendrogram = c("row"))

# upload csv file of dataset : ITS2 mitogenome variability

View(ITS2)

x <- read.csv("ITS22.csv", row.names = 1)

y <- data.matrix(x)

#set color palette

my_palette <- colorRampPalette(c("yellow", "red"))(n = 50)

#create heatmap

heatmap.2(y, main = "ITS2", trace = "none",

density.info=c("none"),

margins = c(5,5),

labRow = NULL,

notecex = 1.0,

notecol = "black",

keysize = 1.5,

col =my_palette, dendrogram = c("row"))
