# Supplementary material for: De novo assembled mitogenome analysis of Trichuris trichiura from Korean individuals using nanopore-based long-read sequencing technology
Source: PLoS Negl Trop Dis. 2023 Aug 28;17(8):e0011586. doi: 10.1371/journal.pntd.0011586 (PMC10491297; doi:10.1371/journal.pntd.0011586)
Supplement: S2 File — (DOCX) [file pntd.0011586.s006.docx]

**S2 File. List of sequences included in the analysis.**

| Species | Host | Sequence type | Accession number | Country |
| --- | --- | --- | --- | --- |
| *Trichuris trichiura* | *Homo sapiens* (Humans) | Complete mitogenome | JF690962 | Czech Republic |
|  | *Homo sapiens* (Humans) | Complete mitogenome | FR846241 | Spain |
|  | *Homo sapiens* (Humans) | Complete mitogenome | AP017704 | Japan |
|  | *Homo sapiens* (Humans) | Complete mitogenome | KT449826 | Uganda |
|  | *Homo sapiens* (Humans) | Complete mitogenome | ON646012 | Korea |
|  | *Homo sapiens* (Humans) | Complete mitogenome | ON711246 | Korea |
|  | *Homo sapiens* (Humans) | Complete mitogenome | ON682760 | Korea |
|  | *Homo sapiens* (Humans) | Complete mitogenome | GU385218  ERS6157129-38 | China |
|  | *Homo sapiens* (Humans) | *cox1* region | ERS6157144-52 | Cameroon |
|  | *Homo sapiens* (Humans) | *cox1* region | ERS6157190-92 | Denmark |
|  | *Homo sapiens* (Humans) | *cox1* region | ERS6157221-33 | Honduras |
|  | *Homo sapiens* (Humans) | *cox1* region | ERS6157237-47 | Tanzania |
|  | *Homo sapiens* (Humans) | Complete mitogenome | KT449826  ERS6157246-67 | Uganda |
|  | *Homo sapiens* Ancient DNA | Complete mitogenome | KY368769 | Denmark |
| *Trichuris sp.* | *Colobus guereza kikuyensis* (Colobus monkey) | *cox1* region | HE653116  ERS6157210-11 | Spain |
|  | *Papio papio*  (Guinea baboon) | Complete mitogenome | MW448472 | Spain |
|  | *Papio anubis*  (Olive baboon) | Complete mitogenome | JF690964 | Czech Republic |
|  | *Papio hamadryas*  (Hamadryas baboon) | Complete mitogenome | JF690963 | Czech Republic |
|  | *Macaca fascicularis*  (Longtailed macaque) | Complete mitogenome | JF690967 | Czech Republic |
|  | *Trachypithecus francoisi*  (Leaf monkey) | *cox1* region | ERS6157139-42 | China |
| *Trichuris suis* | *Sus scrofa domestica* (Domestic pig) | Complete mitogenome | HE653124 | Spain |
|  | *Sus scrofa domestica* (Domestic pig) | Complete mitogenome | HQ204210 | China |
|  | *Sus scrofa scrofa*  (Wild boar) | Complete mitogenome | HE653127 | China |
| *Trichuris muris* | *Mus domesticus*  (House mouse) | Complete mitogenome | HE653130 | Spain |
| *Trichuris arvicolae* | *Myodes glareolus*  (Bank vole) | Complete mitogenome | FR851284 | Spain |
| *Trichuris vulpis* | *Canis lupus familiaris*  (Dog) | Complete mitogenome | HE653138 | Spain |
| *Trichuris skrjabini* | *Capra hircus*  (Domestic goat) | Complete mitogenome | HQ183745 | China |
| *Trichuris ovis* | *Capra hircus*  (Domestic goat) | Complete mitogenome | MG837081 | China |
| *Trichuris discolor* | *Bos taurus*  (Cattle) | Complete mitogenome | HE653139 | Spain |
| *Trichinella spiralis (outgroup)* | - | Complete mitogenome | NC_002681.1 | USA |
