# Supplementary material for: De novo assembled mitogenome analysis of Trichuris trichiura from Korean individuals using nanopore-based long-read sequencing technology
Source: PLoS Negl Trop Dis. 2023 Aug 28;17(8):e0011586. doi: 10.1371/journal.pntd.0011586 (PMC10491297; doi:10.1371/journal.pntd.0011586)
Supplement: Attachment — Submitted filename: Delaluna et al, 2023.docx [file pntd.0011586.s007.docx]

In the study by Delaluna and colleagues entitled “De novo  assembled mitogenome analysis of *Trichuris* trichiura from Korean individuals using nanopore-based long-read sequencing technology”, the authors characterized whole mitochondrial genomes of three human *Trichuris*, *Trichuris trichiura*, isolates from 3 human patients in South Korea using long read sequencing of Nanopore. The authors analyzed the mitochondrial genomes using variants characterization and phylogenetic analysis with other published mitochondrial genomes.

The manuscript represents a good resource for parasite genomics and diagnostics since it is the first study to characterize mitochondrial genomes of human *Trichuris* using long read sequencing which had an advantage of characterizing the genome with high accuracy (e.g. precise estimation of AT region). The methods are suitable and the conclusions are supported by the results. Although the overall conclusion is not novel, it supports the potential existence of two species of human *Trichuris*.

I have only a few suggestions.

In Figure 6, the authors identified 3 clusters that separate *Trichuris* from 3 hosts, humans, NHP and pigs. There are more rrnL sequences from NHP that suggest it is not differentiable from human *Trichuris*. I suggest the authors to include more sequences of rrnL from NHP and make more comprehensive tree that reflects the complexity of the *Trichuris* phylogeny.

Also, the authors used cox1 and rrnL when exploring the relatedness between *Trichuris* from different species. However, both markers are conserved markers. I suggest to also include other variable markers such as nad1 to give a phylogenetic relationship from a different perspective that may resolve a potential hidden sub-cluster of *Trichuris*.

One minor comment, I encourage the authors to put the versions of the used R packages and R version and other used software.
