# Supplementary material for: De novo assembled mitogenome analysis of Trichuris trichiura from Korean individuals using nanopore-based long-read sequencing technology
Source: PLoS Negl Trop Dis. 2023 Aug 28;17(8):e0011586. doi: 10.1371/journal.pntd.0011586 (PMC10491297; doi:10.1371/journal.pntd.0011586)
Supplement: Attachment — Submitted filename: Response to reviewers_H Song.docx [file pntd.0011586.s008.docx]

July 21, 2023

Bruce A. Rosa

Academic Editor

PLOS Neglected Tropical Diseases

Cinzia Cantacessi

Section Editor

PLOS Neglected Tropical Diseases

Re: Revision requested for YEXER_2020_147R1

Dear Dr. Rosa and Dr. Cantacessi:

We would like to thank editors and reviewers of the *PLOS Neglected Tropical Diseases* for taking their time to review our manuscript entitled “**De novo assembled mitogenome analysis of *Trichuris trichiura* from Korean individuals using nanopore-based long-read sequencing technology**”.

Having gone over the suggested comments, we have made appropriate corrections and clarifications. We appreciated to all reviewers that they pointed out most important and critical points to improve our data. We have carried out the experiments that the reviewers suggested, responded to all comments of reviewers, revised the text, and added new descriptions in the text according to the reviewer’s comments.

Following is the summary of revision points. Revised portions were marked in the text by highlights. Each of the coauthors has seen and agreed with the changes made to this revision. I hope the revised manuscript will better meet the publication requirement of the *PLOS Neglected Tropical Diseases*.

Thank you for your kind reconsideration in advance and I am looking forward to hearing a positive reply from you.

Sincerely,

Hyun Beom Song

- Hyun Beom Song, MD, PhD: Department of Parasitology and Tropical Medicine, Seoul National University College of Medicine, 28 Yongon-dong Chongno-gu, Seoul 110-744, Republic of Korea

Tel: 82-2-740-8341 Fax: 82-2-765-6142 E-mail: hbsong@snu.ac.kr

**Reviewer #1:**

In the study by Delaluna and colleagues entitled “*De novo* assembled mitogenome analysis of *Trichuris trichiura* from Korean individuals using nanopore-based long-read sequencing technology”, the authors characterized whole mitochondrial genomes of three human *Trichuris*, *Trichuris trichiura*, isolates from 3 human patients in South Korea using long read sequencing of Nanopore. The authors analyzed the mitochondrial genomes using variants characterization and phylogenetic analysis with other published mitochondrial genomes.

The manuscript represents a good resource for parasite genomics and diagnostics since it is the first study to characterize mitochondrial genomes of human *Trichuris* using long read sequencing which had an advantage of characterizing the genome with high accuracy (e.g. precise estimation of AT region). The methods are suitable and the conclusions are supported by the results. Although the overall conclusion is not novel, it supports the potential existence of two species of human *Trichuris*.

I have only a few suggestions.

In Figure 6, the authors identified 3 clusters that separate *Trichuris* from 3 hosts, humans, NHP and pigs. There are more rrnL sequences from NHP that suggest it is not differentiable from human *Trichuris*. I suggest the authors to include more sequences of rrnL from NHP and make more comprehensive tree that reflects the complexity of the *Trichuris* phylogeny.

Author response:

We appreciate the Reviewer’s recommendation. We were able to incorporate additional rrnL sequences from NHP and other isolates of *Trichuris* sp to make a robust phylogenetic analysis. Previously we used 22 *rrnL* sequence (including outgroup), 2 of which are NHP sequences. During the revision, we now have 98 sequences (including outgroup), where we added 32 more NHP sequences (shown below as red text in the raw phylogenetic tree constructed)

We have collapsed most of the taxa clustering in the tree presented in our results (Lines 433-457) to better visualize the relationship among taxa.

Also, the authors used cox1 and rrnL when exploring the relatedness between *Trichuris* from different species. However, both markers are conserved markers. I suggest to also include other variable markers such as nad1 to give a phylogenetic relationship from a different perspective that may resolve a potential hidden sub-cluster of *Trichuris*.

Author response:

We are thankful for insightful comment, and we agree with the importance of using highly variable markers to produce a better resolved phylogenetic tree. During the revision, we extracted nd*1* region of whole mitochondrial sequences from the database and generated a phylogenetic tree.

Using *nd1* as a mitogenome marker we produced this tree that provide a better resolution of the intraspecies relationship among human derived *T. trichiura* sequences among different geographical regions. We could identify 3 groups in human *Trichuris* and two possible subgroup in one of the groups.

We have collapsed most of the taxa clustering in the tree presented in our results (Lines 531-549) to better visualize the relationship among taxa.

One minor comment, I encourage the authors to put the versions of the used R packages and R version and other used software.

Author response:

We have added the R version of the software we used. Please see lines 192 and 196.

“Pairwise comparison was performed in Geneious prime whereas concatenated protein coding genes (PCGs), *cox1*, and *rrnS* regions of each mitogenome were extracted and aligned with the corresponding reference sequences to report variability and create a heatmap using R studio (version R.4.1.2). Also, all mitogenomes were aligned to each other and compared using *dnadiff* in the MUMmer package (25, 26). Then, principal component analysis (PCA) was performed using average nucleotide identity (ANI) of each pair of mitogenomes. Results were tabulated to produce a PCA graph in R studio (version R.4.1.2). All scripts for each step are compiled in the supplementary file (S1 File).”

**Reviewer #2:**

The manuscript by Delaluna and colleagues describes mitochondrial genome of Trichuris trichiura from Korean individuals using nanopore-based long-read sequencing. Overall, the article is well written and the findings are of interest to scientists interested in evolutionary aspects and systematics of this and closely related moths. I do find the experimental approach overall adequate; however, one major concern relates to the novelty of this work. Therefore, the impact of paper is not so that it can be published in this high-quality journal. I strongly recommend authors to find an alternative journal for their work.

Author response:

We understand the reviewer’s concerns and we also honor this high-quality journal. However, we have applied long reads that can provide a good resource for parasite genomics and diagnostics for future experiments and comparisons. In addition, we wanted to provide cost-effective methods for mitogenome analysis with detailed information for researchers in STH-endemic countries who have samples but without much resource. We can expect higher impact if we can have a chance to publish in this high-quality journal.
